# Comparison of intra subject repeatability of quantitative fluoroscopy and static radiography in the measurement of lumbar intervertebral flexion translation

**DOI:** 10.1038/s41598-019-55905-1

**Published:** 2019-12-17

**Authors:** Alexander Breen, Emilie Claerbout, Rebecca Hemming, Ravi Ayer, Alan Breen

**Affiliations:** 1 Centre for Biomechanics Research, AECC University College, Parkwood Rd, Bournemouth, BH5 2DF UK; 2 Arthritis Research UK Biomechanics and Bioengineering Centre, School of Healthcare Sciences, Cardiff University, Eastgate House 35 - 43 Newport Road, Cardiff, CF24 0AB UK; 3 Radiology Department, Poole General Hospital NHS Foundation Trust, Longfleet Rd, Poole, BH15 2JB UK; 4 Faculty of Science and Technology Bournemouth University, Fern Barrow, Poole, BH12 5BB UK

**Keywords:** Computational biophysics, Diagnostic markers

## Abstract

Low back pain patients are sometimes offered fusion surgery if intervertebral translation, measured from static, end of range radiographs exceeds 3 mm. However, it is essential to know the measurement error of such methods, if selection for back surgery is going to be informed by them. Fifty-five healthy male (34) and female (21) pain free participants aged 21–80 years received quantitative fluoroscopic (QF) imaging both actively during standing and passively in the lateral decubitus position. The following five imaging protocols were extracted from 2 motion examinations, which were repeated 6 weeks apart: 1. Static during upright free bending. 2. Maximum during controlled upright bending, 3. At the end of controlled upright bending, 4. Maximum during controlled recumbent bending, 5. At the end of controlled recumbent bending. Intervertebral flexion translations from L2-S1 were determined for each protocol and their measurement errors (intra subject repeatability) calculated. Estimations using static, free bending radiographic images gave measurement errors of up to 4 mm, which was approximately twice that of the QF protocols. Significantly higher ranges at L4-5 and L5-S1 were obtained from the static protocol compared with the QF protocols. Weight bearing ranges at these levels were also significantly higher in males regardless of the protocol. Clinical decisions based on sagittal translations of less than 4 mm would therefore require QF imaging.

## Introduction

Low back pain is responsible for the world’s largest number of days lost to disability^1^ and although its diagnosis is often problematical, it is agreed that mechanics generally, and segmental stability in particular, plays a significant role^2–4^. However, the measurement of segmental stability in patients is problematical due to lack of a unified concept of the condition. Yet while biomechanical measurements alone are not considered to be good predictors of prognosis, patients with sufficiently severe symptoms may be offered fusion surgery if intervertebral translation exceeds 4 mm^5^. There are many imaging methods for determining this, but practicality and economics dictates that it is generally performed using standing end-range radiographs^6^.

For this measurement, a radiograph is taken in the neutral standing position and then with the patient flexing forward as far as possible. This is repeated with the patient bending backwards into extension. On the resulting images lines are drawn on adjacent vertebrae from which to measure the translation or sliding movement between vertebrae. This is generally preferred by clinicians to angular movement for the assessment of stability^7^. However, it has long been recognised that inaccuracies and population variations using this technique may limit its usefulness and make selection of a cut off for excessive translation difficult^8^. Static views have also been found to underestimate intervertebral translation compared to dynamic imaging and the lateral decubitus position to better detect excessive motion in spondylolisthesis cases^9,10^. Furthermore, complexity increases if the patient also has spinal stenosis^11^ or if revision surgery is being considered^12^.

Recently, advances in fluoroscopic imaging have made it possible to register and track multi segmental vertebral image sequences throughout the entire motion. This method is called quantitative fluoroscopy (QF) and has been able to identify motion patterns that discriminate patients with chronic, nonspecific back pain from pain free controls^13–17^. It has also been used to measure positional changes at individual levels, where for translation, it has been reported to have an accuracy of 0.1 mm and inter-observer repeatability of 1.1 mm (agreement) and ICC 0.533–0.988 (reliability)^18,19^. Given the ubiquity of fluoroscopes in general hospitals, these might be repurposed to provide an alternative method for measuring inter vertebral translation in such patients.

**Figure 1:**
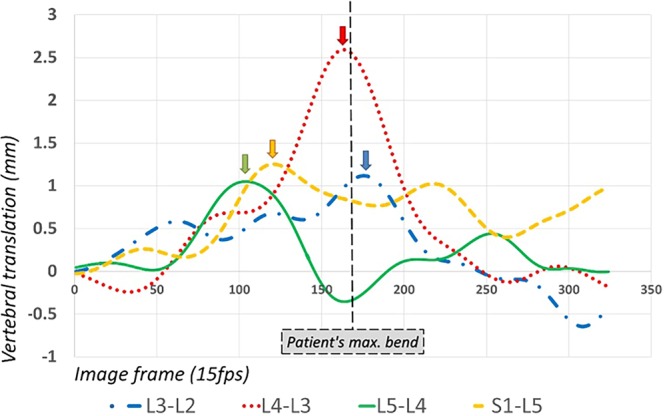
Example of continuous translational motion from L2-S1 in a healthy control participant showing the points of maximum translation (coloured arrows) compared to the point of the patient’s maximum trunk bend.

Continuous standardised motion measurement has a number of potential advantages. First, although the motion is not ‘naturally performed’, controlled motion enables standardisation for trunk range, velocity, ramp up and ramp down speeds and is therefore potentially more reproducible. Second, QF can be conducted either actively weight-bearing or passively in recumbence, to avoid muscle contraction, or guarding, and to test the passive structures with minimal uncontrolled movement variation^20^. Third, the option of a passive recumbent examination has the advantage of additional patient comfort, where upright bending may be inhibited by pain. Fourth, the range of translation may be measured at the end of the maximum range of the segment, which may not coincide with its range at the end of the trunk bending motion (Fig. 1).

As QF allows for a number of protocols for measuring intervertebral translation it was thought useful to assess the measurement properties of these in terms of random and intra subject variability for measuring maximum displacement. In addition, a direct comparison of end range vs through range translation is lacking, as is measurement during free and guided bending^21^. The main aim of this study was to compare the intra subject variability, or measurement error, of 5 methods for measuring intervertebral flexion translation to determine the level of difference that could be detected by each. The evaluation of extension was not included as the standing range of lumbar spine extension is small (20°)^22^.

## Methods

### Participants

Fifty-five healthy control participants were recruited from staff, students and visitors of the AECC University College (Bournemouth, UK). To be eligible, participants had to be aged 21–80 years, BMI < 30, with no history of previous back or abdominal surgery or spondylolisthesis, no medical radiation exposure of >8 mSV in the previous 2 years and no current pregnancy. Participants also had to have been free of any back pain that limited their normal activity for more than 1 day in the previous year. All imaging was carried out in accordance with AECC UC Local Rules and ethical approval was obtained from the National Research Ethics Service (South West 3, 10/H0106/65). Written Informed consent was obtained from all individual participants included in the study. All images of models were submitted with the express permission and signed informed consent of the model for publication of identifying information/images in an online open-access publication.

### Data collection

**Figure 2:**
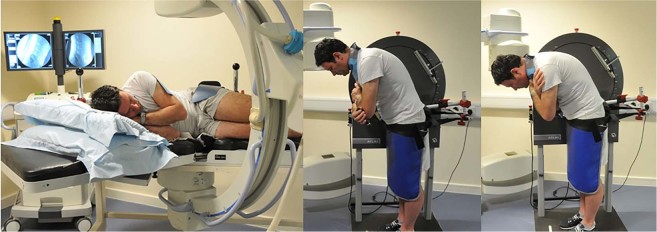
Dynamic acquisition of fluoroscopy sequences: (**a**) controlled passive recumbent flexion, (**b**) controlled active weight bearing flexion, (**c**) uncontrolled weight bearing flexion.

Participants (median age 30 years, range 21 to 69), received fluoroscopic imaging of their lumbar spines during both lying (passive recumbent) and standing (weight-bearing guided) flexion. In passive recumbent flexion. For passive imaging they lay unconstrained in the lateral decubitus position on a motorised table that flexed their upper body to 40° flexion and return during fluoroscopic screening (Atlas Clinical Ltd.) (Fig. 2a). They were then imaged whilst weight-bearing, standing with their right side against the motion frame using the same controller apparatus as for the recumbent procedure (Fig. 2b).

**Figure 3:**
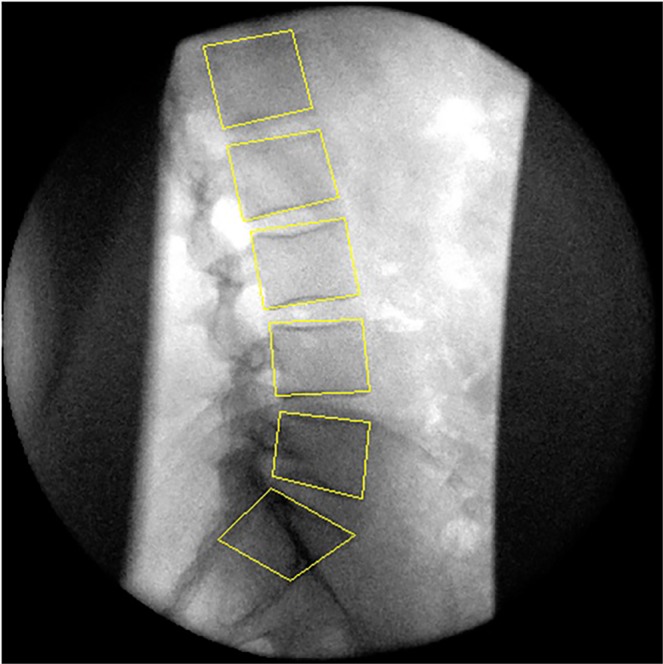
Sagittal lumbar spine fluoroscopic image showing computer reference templates.

With their pelvises stabilised and during active voluntary motion, participants were guided through a standardised range of 60° standing flexion and return by a moving arm. The motion controllers accelerated at 6°s^−2^ for the first second followed by a uniform 6°s^−1^ thereafter. The guiding arm was then removed, and the participants were asked to bend forward freely to the end of their comfortable range (weight-bearing unguided flexion) (Fig. 2c). Single fluoroscopic images were obtained at the beginning and end of the weight-bearing unguided flexion motion. Fluoroscopic motion sequences were recorded at 15 Hz using a Siemens Arcadis Avantic digital C-arm fluoroscope (Siemens GMBH) and stored in DICOM format. They were then exported to a computer workstation and analysed using manual first image registration (Fig. 3) and thereafter using bespoke frame-to-frame tracking using codes written in Matlab (V2011a, The Mathworks Inc). These measurements were repeated 6 weeks later by the same operator using the same equipment at approximately the same time of day for the determination of intra-subject measurement error^21^.

### Image analysis

Sagittal plane translation was calculated using the method of Frobin *et al*. in vertebral body units (VBU) which were converted to millimetres for presentation by multiplying the result by 35 - the standard chosen for vertebral body depth in millimetres^23^. In order to address the degree of translation that could be considered excessive, sagittal plane translation of each intervertebral level from L2-S1 was determined and the levels pooled to provide means and upper reference ranges of variation (+1.96 SD) for the following five measurement protocols:

1. Maximum IV translation during passive recumbent flexion
2. IV translation at maximum bend of passive recumbent flexion
3. Maximum IV translation during guided weight-bearing flexion
4. IV translation at maximum bend of guided weight-bearing flexion
5. IV translation at maximum bend of unguided weight-bearing flexion (reflective of traditional static radiograph acquisition)

### Statistical analysis

All data were tested for normality using the Shapiro-Wilk test. The significance of differences was calculated using 2-way paired t-tests for normally distributed data and the Wilcoxon test for non-normal data. Repeatability was calculated using the following formula, where S_w_ is the within-subject standard deviation. The repeatability coefficient, or measurement error, estimates the magnitude of the within-subject change that can be expected 95% of the time and represents the Minimum Detectable Change (MDC_95_)^21^. Source data for this study] are available by application to the corresponding author.

$${\rm{Repeatability}}\,{\rm{coefficient}}({{\rm{MDC}}}_{95})=2.77{{\rm{S}}}_{{\rm{w}}}$$

The association between test-retest differences and their means were assessed using Kendall’s tau. As no significant and/or substantial associations were found, the data were not transformed for the calculation of MDC_95._

## Results

Fifty-five participants (21F, 34M) were recruited and all provided complete data. These data were mainly distributed non-normally, resulting in a nonparametric approach to statistical comparisons. Participants’ characteristics were: height 1.75 m (range 1.53–1.90), weight 74.9 kg (range 47.6–112.4) and BMI 24.2 (range 16.9–31.8). The median effective dose received per participant was 0.27 mSv for weight bearing motion (range 0.20–0.68), 0.18 mSv for recumbent motion (range 0.11–0.31) and 0.04 mSv for single frame maximum bend images (range 0.01–0.09).

The median translations for pooled L2-S1 levels were less than 2 mm regardless of protocol while the static uncontrolled protocol gave significantly higher translation ranges than any of the controlled protocols (p < 0.001, (Wilcoxon) (Table 1). Intra class correlations were moderate to substantial, showing acceptable reliability for all protocols, however, the measurement error was highest (3.36 mm) for the static uncontrolled protocol, compared with the highest error of the controlled protocols (2.14 mm). This reflects an error in excess of 200% of the baseline translation for the static protocol compared with a maximum of 163% for controlled weight bearing. Weight bearing measurements, both guided and unguided, gave slightly higher ranges than passive recumbent testing, but similar values when measured at the end of the motion and during it.

**Table 1:** Translation ranges, reliability and measurement error for five measurement protocols (L2-S1 pooled data).

Measurement	Protocol	n	Median translation (IQR) mm		Reliability	Measurement error
			Baseline	Follow up	ICC_2,1_ (95% CI)	MDC_95_
During motion 40 deg	Passive recumbent	219	0.74 (0.69)	0.86 (0.78)	0.639 (0.528, 0.724)	1.33
End of motion 40 deg	Passive recumbent	219	0.74 (0.58)	0.86 (0.53)	0.611 (0.486, 0.706)	1.43
During motion 60 deg	Active weight bearing	216	1.21 (1.26)	1.21 (1.37)	0.550 (0.413, 0.655)	1.97
End of motion 60 deg	Active weight bearing	216	1.22 (1.05)	1.31 (1.08)	0.782 (0.715, 0.833)	2.14
End of uncontrolled flexion	Active weight bearing	200	1.54 (1.42)	1.47 (1.67)	0.697 (0.605, 0.768)	3.36

**Figure 4:**
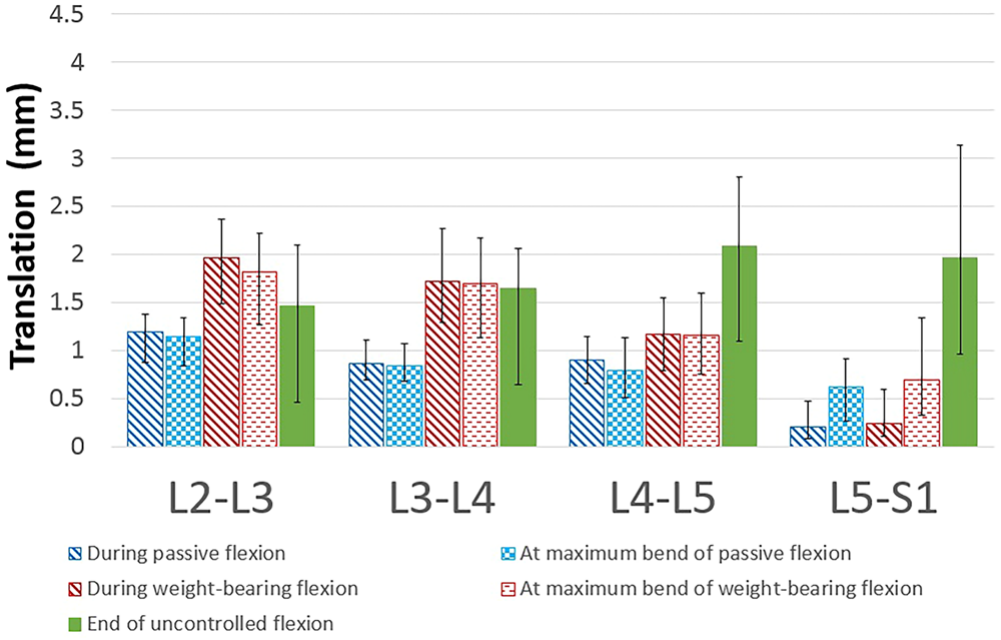
Median baseline translations (interquartile range) for each level from L2-S1 for five measurement methods.

When taken level by level, the median baseline translation of L2-3 was significantly greater during guided weight bearing continuous measurement than unguided weight bearing static measurement (p < 0.001), whereas the converse was true for L4-5 and L5-S1 (p < 0.001) (Wilcoxon) (Fig. 4).

**Figure 5:**
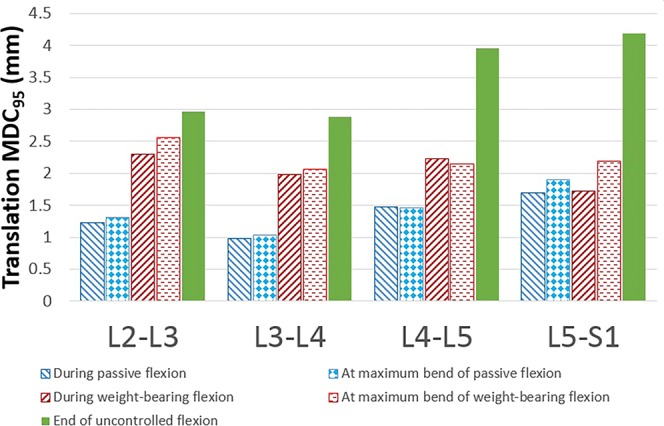
Measurement error (MDC_95_) for translations for each level from L2-S1 for five measurement methods.

The measurement errors at L4-5 and L5-S1 for static uncontrolled measurements at around 4 mm were approximately double those of controlled ones (around 2 mm), however, for L2-3 and L3-4 these differences were less marked (Fig. 5).

**Figure 6:**
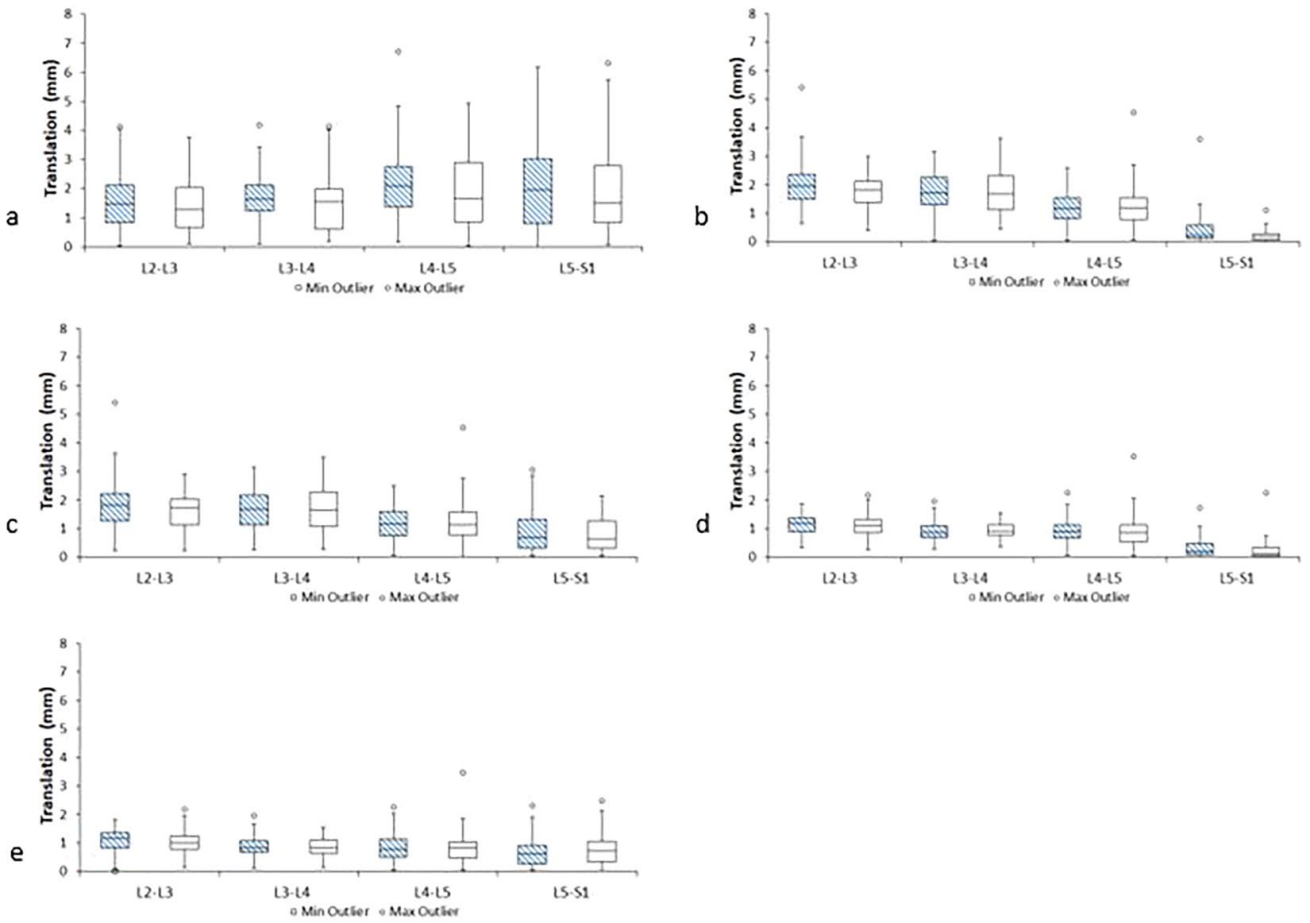
Box plots showing median intervertebral translations from L2-5 at baseline (hatched box) and follow-up (clear box) measured (**a**) at end of uncontrolled weight bearing flexion (**b**) during controlled weight bearing flexion (**c**) at end of controlled weight bearing flexion (**d**) during recumbent flexion (**e**) at end of recumbent flexion.

The baseline median translation range at L5-S1, when measured using the static, uncontrolled maximum bend protocol, was significantly higher for males than for females (P < 0.001) (Mann Whitney). In addition, for the weight bearing controlled bending protocols, L3-4 and L4-5 ranges were higher for males (p < 0.01), while recumbent sequences measured during the motion gave higher ranges at L2-3 for females (p < 0.05). Age above and below the inter-quartile ranges did not have any significant effect on translation range for any level or protocol. It should also be noted that L5-S1 translation, measured using controlled motion protocols, returned very small values when measured during motion as opposed to at its end, while weight bearing measurements returned more variation and less consistency than recumbent ones (Fig. 6a–e).

## Discussion

This research found that static radiographs gave twice the measurement error of QF and higher L4-5 and L5-S1 ranges when used to measure flexion translation. In effect, this means that it is not possible to detect translation of under 4 mm using static radiographs, 2.5 mm using weight bearing QF or 2.0 mm using recumbent QF. Furthermore, the normative ranges for each protocol are different for males and females, but not in older people. A cut-off at 4 mm for inferring instability is consistent with much of the literature as reviewed by Leone *et al*., however, as recognised by Nizard *et al*., population variation and lack of standardisation have made any such cut off somewhat tenuous^5,24^. Nevertheless, Posner *et al*.’s criterion for selecting patients with instability for fusion treatment, which defines a cut off of 8% of vertebral body depth for anterior translation is generally accepted, although this would amount to only 2.8 mm using a standard intervertebral body depth of 35 mm^25–27^.

In this study, measurement at the end of uncontrolled motion using static radiographs was more variable than using QF. At L4-5 and L5-S1, this returned approximately twice the measurement error of the QF protocols, while static, uncontrolled weight bearing measurements were similar to guided weight bearing QF measurements at L2-3 and L3-4.

The least population variability and measurement error was found when participants were imaged during passive recumbent motion, as has been recommended for the detection of excessive translation in spondylolisthesis^10^.

The 4 mm measurement error for weight bearing, static, unguided, end of range measurements was especially applicable to L4-5 and L5-S1. These levels are frequently of interest in terms of translatory slip, however, this may be uncommon in back pain populations. A recent study of aberrant motion in chronic, nonspecific back pain did not find translation to be greater in patients than healthy controls^17^. Even in patients with spondylolisthesis, excessive translation is also not necessarily a feature, while in older individuals with degenerative spinal stenosis, bone loss, arthritic outgrowth and vertebral mal-alignment may make the measurement of translation using any current form of radiographic imaging additionally problematical^5,28^.

The tendency for static views, acquired at the end of trunk motion, to give different values from QF may be thought to be because the range of trunk motion at the end of a weight-bearing unguided flexion motion could be greater than 60°, which is the standard range of flexion used for standing guided weight-bearing QF^19^. However, free bending resulted in only approximately 0.5 mm greater translation than controlled bending to 60°. Indeed, the median ranges of translation found in this study, by all of the protocols, compare favourably with those found in a separate study of healthy volunteers^29^. However, although studies of intervertebral translation in back pain patients have concluded that it is related to age and disc height, it does not differentiate patients from controls^17,30,31^. This may be partially a result of the uncontrolled variation associated with current measurement methods. However, composite disc degeneration throughout the lumbar spine has been associated with disproportionate sharing of angular motion between the lumbar spine segments in chronic, nonspecific back pain patients^16^. Thus, it may be that it is the distribution of degenerated discs in the lumbar spine, rather than large changes in ranges of motion at individual levels, that is most closely associated with symptoms in chronic, nonspecific low back pain^32^.

Finally, the qualitative use of fluoroscopy tends to be associated with prolonged exposures, raising the expectation of higher radiation dosage. However, the QF protocols are, by definition, quantitative and in this study resulted in effective radiation dosages of less than 0.3 mSv each. This is considerably less than the 1.3 mSv quoted as the typical effective dose expected for a series of X-rays of the lumbar spine for diagnostic purposes^33,34^. This makes continued of the use of plain radiographs difficult to justify for most cases where degrees of increased translation that are not measurable might be acted upon.

### Limitations

The present study did not include extension motion; however, its purpose was to compare radiographic techniques for their measurement properties while minimising radiographic exposure. The levels considered also did not include L1 because the intensifier diameter was too small to permit it.

### Further work

These methods, although tested on a healthy asymptomatic population here, have also been utilised to evaluate back pain populations^16,17^. Therefore, this study should be repeated in symptomatic cohorts to establish repeatability and variability of translation.

## Conclusion

Quantitative fluoroscopic measurement of lumbar intervertebral flexion translation in healthy control participants during passive recumbent QF gave significantly lower values than static, weight-bearing unguided imaging. It also resulted in lower population variation and approximately half the measurement error, which for static images during uncontrolled motion was in the region of 4 mm. Thus, clinical decisions based on smaller amounts of sagittal translation would require QF imaging.
